# Genomic epidemiology of SARS-CoV-2 in large university hospital cohort: the UnCoVER-Brazil project

**DOI:** 10.1017/S095026882300119X

**Published:** 2023-07-20

**Authors:** Felipe Santos de Carvalho, Sarah Danielle Slack, Francisco Barbosa-Júnior, Mateus Rennó de Campos, Guilherme Silveira Castro, Sabrina Baroni, Livia Mara Torres Bueno, Fernanda Borchers Coeli, Aparecida Yulie Yamamoto, Jorgete Maria Silva, Rodrigo do Tocantins Calado, Benedito Antônio Lopes Fonseca, Leandro Machado Colli, Fernando Bellissimo-Rodrigues

**Affiliations:** 1 Social Medicine Department, Ribeirão Preto Medical School, University of São Paulo, Ribeirão Preto, Brazil; 2 Infectious Diseases Division, Internal Medicine Department, Ribeirão Preto Medical School, University of São Paulo, Ribeirão Preto, Brazil; 3 Medicine Department, Federal University of São Carlos, São Carlos, Brazil; 4 Oncology and Imaging Department, Ribeirão Preto Medical School, University of São Paulo, Ribeirão Preto, Brazil; 5 Epidemiological Surveillance Service, University Hospital of Ribeirão Preto Medical School, University of São Paulo, Ribeirão Preto, Brazil; 6 Virology Laboratory, University Hospital of Ribeirão Preto Medical School, University of São Paulo, Ribeirão Preto, Brazil

**Keywords:** COVID-19, epidemiology, genome, SARS-CoV-2, variants

## Abstract

This work aimed to study the role of different SARS-CoV-2 lineages in the epidemiology of multiple waves of the COVID-19 pandemic in Ribeirão Preto (São Paulo state), with comparison within Brazil and globally. Viral genomic sequencing was combined with clinical and sociodemographic information of 2,379 subjects at a large Brazilian hospital. On the whole 2,395 complete SARS-CoV-2 genomes were obtained from April 2020 to January 2022. We report variants of concern (VOC) and interest (VOI) dynamics and the role of Brazilian lineages. We identified three World Health Organization VOCs (Gamma, Delta, Omicron) and one VOI (Zeta), which caused distinct waves in this cohort. We also identified 47 distinct Pango lineages. Consistent with the high prevalence of Gamma in Brazil, Pango lineage P.1 dominated infections in this cohort for half of 2021. Each wave of infection largely consisted of a single variant group, with each new group quickly and completely rising to dominance. Despite increasing vaccination in Brazil starting in 2021, this pattern was observed throughout the study and is consistent with the hypothesis that herd immunity tends to be SARS-CoV-2 variant-specific and does not broadly protect against COVID-19.

Severe acute respiratory syndrome coronavirus 2 (SARS-CoV-2), the virus that causes coronavirus disease (COVID-19), remains a global threat despite massive diagnostic testing, isolation, therapies, and vaccines. Emerging SARS-CoV-2 variants of concern (VOCs) continue to challenge these measures with increased transmissibility or virulence, escape of host antibody neutralization, and decreased efficacy of detection, therapeutics, and vaccination [1]. Viral genomic sequencing can improve the understanding of VOCs while providing insight into the dynamics of all circulating SARS-CoV-2 lineages.

Of the over 650 million global cases of COVID-19 reported by the end of December 2022, 36 million cases were reported in Brazil, with deaths approaching 700,000. Almost 180,000 of these cases were reported in Ribeirão Preto, located in São Paulo state [2]. Ribeirão Preto is home to a prominent national medical centre, making it a unique setting for the study of COVID-19 pandemic dynamics with multiple ongoing studies [3]. This ongoing study offers insight into the role of different SARS-CoV-2 lineages in the epidemiology of multiple waves of the COVID-19 pandemic in Ribeirão Preto, with comparison within Brazil and globally.

All patients cared for at the Hospital das Clínicas, Faculdade da Medicina de Ribeirão Preto, Universidade de São Paulo (HCFMRP-USP), were eligible to be included in the *U*ncovering *n*ew *Co*rona*V*irus *E*ncoded *R*amifications in Brazil (UnCoVER-Brazil) study. Nasopharyngeal and/or oropharyngeal samples were collected for over 6,000 patients in outpatient and inpatient settings at HCFMRP-USP between 4 April 2020 and 31 January 2022. The detection of SARS-CoV-2 was performed by multiplex real-time reverse transcription PCR (RT-PCR) targeting N, E, and RDRP genes (Gene Finder™ COVID-19 Plus Real Amp Kit; OSANG Healthcare). Subjects were included in the UnCoVER study with a cycle threshold for SARS-CoV-2 less than or equal to 30. From 2,637 viral samples meeting this criterion, we generated complete SARS-CoV-2 genomes from 2,395 distinct infections of 2,379 patients. Multiple distinct infections from a single individual were required to have a minimum sample collection interval of 60 days. Sequencing was performed at the Laboratory of Translational Oncology at the Hemocentro of Ribeirão Preto, with the Illumina COVIDSeq Test (RUO Version) kit and the included ARTIC Network nCoV-2019 Amplicon Panel (V3). Most samples were sequenced on HiSeq 4000 (Illumina), with some sequenced on MiSeq (Illumina). FASTQ generation was completed in BaseSpace (Illumina) using the FASTQ Generation app (1.0.0). DRAGEN COVID Lineage (version 3.5.10) was used for alignment to SARS-CoV-2 reference genome NC_045512 and for Pango lineage classification (Pangolin software 4.1.2, Pangolin data 1.14). Only genomes that passed Pangolin quality control and had at least 90% non-ambiguous content were included in analysis [4]. All included SARS-CoV-2 genomes are available on GISAID under the identifier EPI_SET_230119wh (doi: 10.55876/gis8.230119wh). Sequencing results were merged with sociodemographic and clinical data in RStudio (2022.07.01), which was also used for analysis and figure generation. REDCap (7.6.3) was used for data storage.

Sociodemographic information and COVID-19 case details were extracted from the HCFMRP-USP COVID-19 surveillance reporting system, with some entries supplemented with information from electronic medical records. Of the 2,395 distinct SARS-CoV-2 infections captured by sequencing, the median age of subjects was 41.0 (IQR 31–56) years, and 1,469 (61.3%) subjects were female. A total of 2,015 cases were symptomatic (84.1%, missing data 15.2%). Less than a third of patients were hospitalized (666 patients, 27.8%), and 176 patients died (7.3%, missing data 17.5%).

We identified four World Health Organization (WHO) VOCs (Gamma, Alpha, Delta, Omicron) and one WHO variant of interest (VOI) (Zeta), four of which (Zeta, Gamma, Delta, Omicron) caused distinct waves in this cohort. Within these waves, we identified 47 distinct Pango lineages, including multiple Brazilian lineages (Figure 1, Table 1).

**Figure 1.**
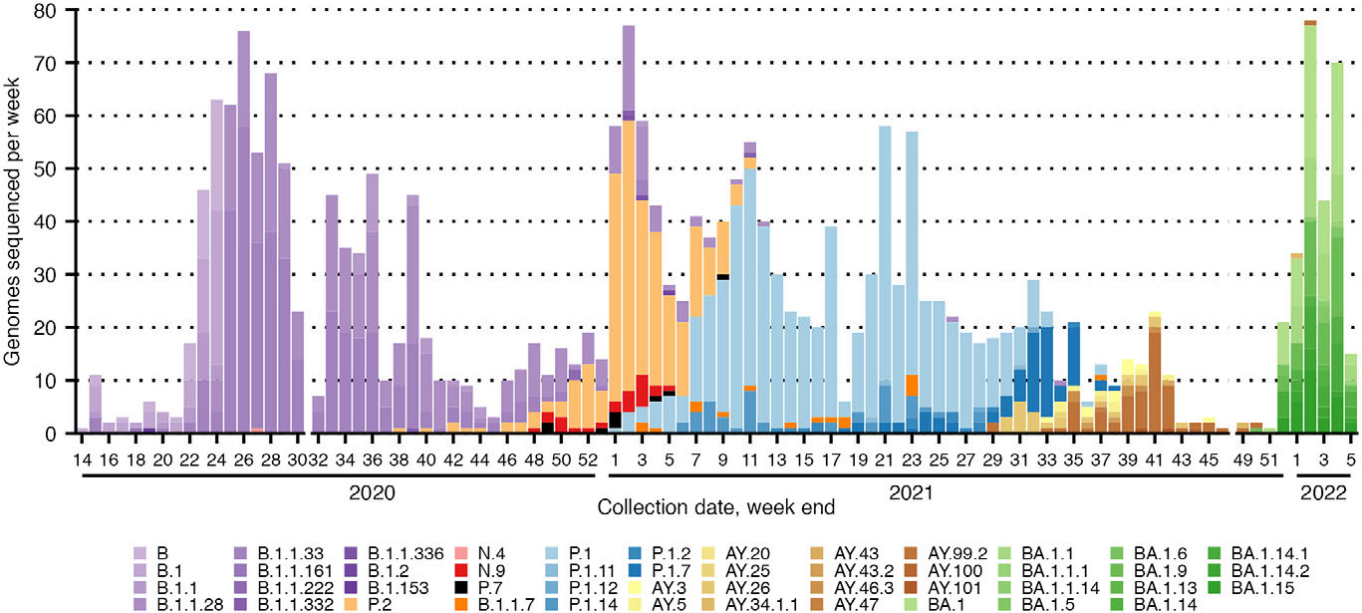
Distribution of SARS-CoV-2 sequenced genomes per epidemiological week, plotted by weekend date (Saturday) of RT-PCR test collection [5]. Bars coloured by SARS-CoV-2 Pango lineage and grouped by VOC, where possible [6]: B & B descendant lineages in shades of purple, Zeta (P.2) in light orange, N lineages in shades of red, non-VOC P lineage in black, Alpha (B.1.1.7) in dark orange, Gamma (P.1) and descendant lineages in shades of blue, Delta (AY lineages) in shades of yellow to brown, and Omicron (BA lineages) in shades of green.

Throughout 2020, the SARS-CoV-2 B lineage and descendants made up nearly all cases sequenced, with Zeta rising at the end of 2020. Gamma spiked early in 2021 and, within 2 months, dominated most sequenced genomes. Gamma continued as the dominant variant until Delta emerged in the last third of 2021. Delta was completely replaced by the Omicron spike in early 2022. Each new dominant variant group quickly and nearly completely replaced the dominant group preceding it (Figure 1). This pattern, which continued despite vaccination increasing in Brazil starting in 2021, is consistent with the hypothesis that herd immunity is SARS-CoV-2 variant-specific and does not broadly cover COVID-19 [7].

We focused our analysis on comparative outcomes between variant groups. Relative to the overall median age of 41.0 years and the largely similar variant group median ages (Table 1), a decrease to 35.0 and 33.0 was observed for N lineages and the non-VOC P lineage, respectively. However, interpretation is limited by the low number of cases sequenced in these groups. Hospitalization was similarly consistent among variant groups, with an overall average rate of 27.8% and range from 20% to 45% for all groups except Omicron. Less than 10% of individuals with Omicron were hospitalized (Table 1); however, we only captured patients early in its wave, and increased vaccination is a likely confounder for decreased severity.

**Table 1.** Identified SARS-CoV-2 Pango lineages, grouped by VOC where possible, with median age and hospitalization counts by variant groups

Pango lineage	*N*	% of all data	% of variant group	Variant group	N	Median age (IQR)	Hospitalized (%)
B	48	2.0	5.3	B & B descendant lineages	906	41.0 (31–56)	232 (25.6)
B.1	65	2.7	7.2				
B.1.1	43	1.8	4.7				
B.1.1.28	359	15.0	39.6				
B.1.1.33	381	15.9	42.1				
B.1.1.161	1	0.0	0.1				
B.1.1.222	2	0.1	0.2				
B.1.1.332	3	0.1	0.3				
B.1.1.336	2	0.1	0.2				
B.1.2	1	0.0	0.1				
B.1.153	1	0.0	0.1				
P.2	274	11.4	100.0	Zeta	274	41.0 (31–55)	62 (22.6)
N.4	1	0.0	4.0	N lineages	25	35.0 (29–54)	6 (24.0)
N.9	24	1.0	96.0				
P.7	9	0.4	100.0	Non-VOC P lineage	9	33.0 (30–52)	4 (44.4)
B.1.1.7	17	0.7	100.0	Alpha	17	43.0 (34–60)	7 (41.2)
P.1	630	26.3	81.4	Gamma	774	43.0 (31–56)	301 (38.9)
P.1.11	3	0.1	0.4				
P.1.12	2	0.1	0.3				
P.1.14	47	2.0	6.1				
P.1.2	14	0.6	1.8				
P.1.7	78	3.3	10.1				
AY.3	11	0.5	8.6	Delta	128	41.5 (31–57)	29 (22.7)
AY.5	8	0.3	6.2				
AY.20	1	0.0	0.8				
AY.25	2	0.1	1.6				
AY.26	24	1.0	18.8				
AY.34.1.1	3	0.1	2.3				
AY.43	1	0.0	0.8				
AY.43.2	2	0.1	1.6				
AY.46.3	6	0.3	4.7				
AY.47	1	0.0	0.8				
AY.99.2	66	2.8	51.6				
AY.100	1	0.0	0.8				
AY.101	2	0.1	1.6				
BA.1	76	3.2	29.0	Omicron	262	41.0 (29.25–55)	25 (9.5)
BA.1.1	39	1.6	14.9				
BA.1.1.1	2	0.1	0.8				
BA.1.1.14	2	0.1	0.8				
BA.1.5	5	0.2	1.9				
BA.1.6	2	0.1	0.8				
BA.1.9	47	2.0	17.9				
BA.1.13	1	0.0	0.4				
BA.1.14	15	0.6	5.7				
BA.1.14.1	30	1.3	11.5				
BA.1.14.2	10	0.4	3.8				
BA.1.15	33	1.4	12.6				
Total	2,395	100	–	–	2,395	–	666

The Pango lineage composition of variant groups offers additional insight into transmission dynamics unique to HCFMRP-USP. Combined, B.1.1.28 and B.1.1.33 made up 80% of the sequences observed in the B & B descendant group that dominated throughout 2020. Of global sequences reported to date, over 75% of all B.1.1.28 lineages and 85% of all B.1.1.33 were reported in Brazil [8]. Both descended from European lineage B.1.1 and were considered primary drivers of the first wave of the pandemic in Brazil. VOI Zeta (P.2) and VOC Gamma (P.1) descended from B.1.1.28, while N.9 – which was present at the same time at HCFMRP-USP but dominated by Zeta – descended from B.1.1.33 [9]. More than half of global lineages attributed to Gamma were observed in Brazil [8]. The long dominance of Gamma at HCFMRP-USP is clear in Figure 1, with over 80% observed being P.1 (Table 1).

All Delta lineages observed in this study were AY lineages, and no B.1.617.2 – the lineage first attributed to the global surge of Delta – was observed. Over 50% of Delta lineages were AY.99.2, a Brazilian lineage with over 95% of all global sequences reported in Brazil [8]. AY.99.2 descended from AY.99, which in turn descended from B.1.617.2. In Brazil, Delta was responsible for a more modest surge of cases than was observed in other countries in the second half of 2021. A recent study from Minas Gerais suggested that despite Delta’s increased transmissibility compared to Gamma – which dominated infections in Brazil when Delta arrived – the over 80% vaccination rate in Brazil at the time may have protected against a surge [10]. Unfortunately, no conclusions can be drawn from this study about case surges, as sequencing did not have any fixed correlation with COVID-19 case count at HCFMRP-USP. Although our data suggest that herd immunity is variant-specific, we speculate that Gamma – which was responsible for a larger outbreak in Brazil than in many countries – may have produced an immune response that was more cross-reactive with Delta than other variants.

In this dispatch, we describe the preliminary insights gained from the large COVID-19 study based at HCFMRP-USP, one of Brazil’s largest public academic hospitals located in Ribeirão Preto, São Paulo state. Here we observed trends in VOC prevalence consistent with those observed in general in Brazil. We also identified the contribution of key Brazilian lineages to pandemic waves. Our results are consistent with the hypothesis that herd immunity to SARS-CoV-2 tends to be variant-specific.

## Data Availability

Data that support the findings of this study are available upon a reasonable request addressed to the corresponding author.
